# PD02 Evolving Telemedicine Perspectives And Capacities To Meet Countrywide Breast Cancer Screening Demands

**DOI:** 10.1017/S0266462325101402

**Published:** 2025-12-29

**Authors:** Pedro Galvan, Jose Ortellado, Santiago Garcia, Maria Teresa Baran, Enrique Hilario

## Abstract

**Introduction:**

Telemedicine capacity has evolved and currently offers excellent tools for fortifying health screening systems, particularly for building a cost-effective screening network for the early diagnosis of breast cancer. In this context, a telemedicine driven approach should be directed toward improving breast cancer screening innovation in underserved hospitals. This study evaluated the results of a telemedicine-driven breast cancer diagnostic network in remote public hospitals in Paraguay.

**Methods:**

This is a descriptive study, where the results using telemedicine-driven breast cancer diagnosis innovations in remote public hospitals were evaluated for their ability to improve accessibility and equity to specialized diagnostic services countrywide. For this purpose, the type and frequency of diagnoses performed through a telemedicine platform was determined.

**Results:**

During the study, a digital telemedicine-driven breast cancer screening network was implemented in 34 hospitals countrywide. The screening procedure involved analyzing a digitally acquired mammogram for masses, calcifications, or areas of abnormal density that may indicate the presence of cancer. The implemented cancer screening network performed 840 tests between October and November 2024. This resulted in the following diagnoses: normal (60%); cyst (33%); and fibroadenoma, macrocalcifications, and abnormal area (7%). If there is a significant suspicion of cancer, then tissue will be removed for a biopsy and examined by a pathologist.

**Conclusions:**

According to our results, the telemedicine-driven breast cancer screening innovation network facilitated a timely, pertinent, high-quality tertiary level diagnostic service with equitable access for patients in remote underserved public hospitals in Paraguay. A widespread use assessment is necessary before this breast cancer screening method can be implemented.

